# Phenotypic and PCR-based analysis of antimicrobial resistance in heat-treated multidrug-resistant *Salmonella* spp. and *Campylobacter jejuni* isolated from raw chicken meat

**DOI:** 10.1016/j.psj.2026.107484

**Published:** 2026-07-23

**Authors:** Hamzah M. Al-Qadiri, Batool Khataybeh, Amal Mayyas, Murad A. Al-Holy, Amin N. Olaimat, Islam Hamad, Nivin Al-Alami, Faris Ghalib Bakri, Hana Zakaria, Mohmmad Al-Qaisi, Tariq N. Alsmairat, George Y. Nijmeh, Rana Al-Akhras, Barbara A. Rasco, Zeinab M.H. Mahasneh

**Affiliations:** a Department of Nutrition and Food Technology, School of Agriculture, The University of Jordan, Amman, 11942, Jordan; b Department of Nutrition and Food Technology, Jordan University of Science and Technology, Irbid, 22110, Jordan; c Department of Pharmacy, Faculty of Health Sciences, American University of Madaba, Madaba, 11821, Jordan; d Department of Clinical Nutrition and Dietetics, Faculty of Applied Medical Sciences, The Hashemite University, Zarqa, 13133, Jordan; e Water, Energy, and Environment Center, The University of Jordan, Amman, 11942, Jordan; f Department of Internal Medicine, Division of Infectious Diseases, School of Medicine, The University of Jordan, Amman, 11942, Jordan; g Infectious Disease and Vaccine Center, The University of Jordan, Amman, 11942, Jordan; h Department of Animal Production, School of Agriculture, The University of Jordan, Amman, 11942, Jordan; i School of Medicine, The University of Jordan, Amman, 11942, Jordan; j College of Agriculture, Life Sciences and Natural Resources, University of Wyoming, Laramie, WY, 82071, USA; k Department of Animal Production and Protection, Faculty of Agriculture, Jerash University, Jerash, Jordan

**Keywords:** Chicken meat, *Salmonella*, *Campylobacter*, Antimicrobial resistance, Heat treatment

## Abstract

Thermal processing is widely applied to control foodborne pathogens; however, methodological approaches for evaluating its impact on antimicrobial resistance (AMR) remain limited. This study establishes an integrated framework combining phenotypic susceptibility testing and PCR-based detection to assess heat-induced changes in multidrug-resistant (MDR) *Salmonella* spp. and *Campylobacter jejuni* isolated from raw chicken meat. Isolates were subjected to controlled thermal treatments (55°C and 60°C) for defined exposure intervals. Phenotypic antimicrobial resistance was evaluated using the standardized Kirby–Bauer disk diffusion method, whereas selected antimicrobial resistance determinants (*bla_TEM_, tet(B), sul2*, C257T mutation in the *gyrA* gene, *tet(O)*, and *bla_OXA-61_*) were detected by conventional PCR using validated primer sets and appropriate positive and negative control strains. The methodological comparison revealed that exposure to 60°C significantly reduced phenotypic resistance across multiple antimicrobial classes, whereas 55°C produced minimal effects. In contrast, PCR assays consistently detected antimicrobial resistance gene (ARG) targets across all treatment conditions, including in samples lacking culturable cells. This divergence highlights differences between culture-dependent and PCR-based approaches for assessing antimicrobial resistance following thermal stress and demonstrates the sensitivity of PCR for detecting ARG targets after heat exposure. The proposed combined phenotypic–molecular workflow provides a reproducible approach for AMR assessment in processed food matrices and demonstrates that phenotypic susceptibility testing and PCR-based detection may provide complementary information when evaluating the effects of thermal treatments on MDR foodborne pathogens. This study contributes a practical methodological framework for evaluating AMR in thermally processed food matrices and highlights the value of integrating phenotypic and molecular approaches when assessing the effects of thermal treatments on MDR foodborne pathogens. However, conventional PCR detects only the presence of amplifiable DNA targets and does not provide information regarding DNA integrity, biological functionality, transcriptional activity, or horizontal transfer potential. Therefore, further studies employing complementary molecular and functional approaches are required to evaluate these characteristics following thermal processing.

## Introduction

Poultry meat is one of the most widely consumed animal-protein sources worldwide and plays a critical role in global food security (Abdullah et al., 2026). However, its highly perishable nature and susceptibility to bacterial contamination throughout production, processing, and distribution make it a significant food safety concern. Consequently, poultry products are frequently implicated in the transmission of foodborne pathogens to humans, particularly when appropriate processing, handling, and cooking practices are not adequately implemented (Abebe et al., 2020; Abdullah et al., 2025). Recent studies have reported an increasing prevalence of multidrug resistance (MDR) and antimicrobial resistance (AMR) among *Salmonella* spp. and *Campylobacter jejuni* isolates, which has been largely attributed to the extensive and, in some cases, inappropriate use of antimicrobial agents in veterinary and agricultural settings (Ford et al., 2023; Al-Qadiri et al., 2025; Mayyas et al., 2025). This phenomenon represents a significant global public health threat, as it undermines the clinical effectiveness of critically important antimicrobial classes, including *β*-lactams, macrolides, tetracyclines, and fluoroquinolones, which are routinely employed in the management of severe bacterial infections, particularly among high-risk populations (Luangtongkum et al., 2009). Antibiotic-resistant *Salmonella* spp. and *Campylobacter* spp. have been recognized among the major foodborne pathogens of global public health concern due to their widespread distribution, increasing resistance trends, and substantial disease burden (Tacconelli et al., 2018).

In the United States, non-typhoidal *Salmonella* is among the leading etiological agents of bacterial foodborne illness (Akil and Ahmad, 2019). Mujahid et al. (2023) reported the detection of *Salmonella* spp. in 8.6% of retail chicken breast samples, with 73.1% of the isolates exhibiting resistance to at least one antimicrobial agent and 48.1% meeting the criteria for MDR, defined as resistance to three or more antimicrobial classes. Data from the Foodborne Diseases Active Surveillance Network (FoodNet) reveal an increasing trend in antimicrobial resistance among *Campylobacter* infections in the United States, with ciprofloxacin resistance rising from 24.5% during 2005-2016 to 29.7% in 2017-2018, and erythromycin resistance increasing from 2.6% to 3.3% over the same interval (Ford et al., 2023). Additionally, Li et al. (2025) documented a progressive reduction in the antimicrobial susceptibility of *C. jejuni* to commonly prescribed antibiotics. This trend has been largely attributed to the widespread use of antimicrobials in both human and veterinary medicine, which imposes selective pressure that promotes the emergence, persistence, and dissemination of MDR *Salmonella* spp. and *C. jejuni*. Consequently, chicken meat may serve as an important reservoir for AMR and MDR foodborne pathogens, particularly Gram-negative bacteria, and is recognized as a significant vehicle for the transmission of these microorganisms and their associated antimicrobial resistance genes (ARGs) (Dierikx et al., 2010; Moawad et al., 2017; da Cunha-Neto et al., 2018; Tacconelli et al., 2018; Ford et al., 2023).

Thermal processing of chicken meat using conventional cooking practices is a critical control measure for ensuring food safety, as heat treatment significantly reduces microbial loads and thereby lowers the risk of foodborne illness (ICMSF, 2005; Qu et al., 2024). However, when thermal treatments are insufficient to inactivate pathogenic microorganisms, sublethal heat exposure may occur, leading to cellular injury and the induction of a viable but non-culturable (VBNC) state, in which bacteria remain viable but cannot be recovered by standard culture-based methods (Zhao et al., 2017; James et al., 2021). The efficacy of thermal processing is governed by the applied time–temperature combination, which determines the extent of microbial inactivation. Mild heat treatments may induce sublethal cellular injury without achieving complete bacterial inactivation, whereas exposure to higher temperatures for sufficient durations results in the effective inactivation of vegetative bacterial cells, including AMR and MDR strains (ICMSF, 2005; Pérez-Rodríguez et al., 2019). Nevertheless, inadequate thermal processing may permit the survival of AMR bacteria in food, thereby facilitating the persistence of resistant populations. Furthermore, ARGs may remain detectable following thermal treatment, either within nonviable bacterial cells or as extracellular DNA released during cell lysis. The persistence of PCR-detectable ARGs in thermally processed foods highlights the need for further investigation to determine their integrity, biological functionality, and potential public health significance (Zhang et al., 2018; Pérez-Rodríguez et al., 2019; James et al., 2021).

Exposure to heat stress can compromise bacterial viability, yet sublethal stresses often trigger genotypic and phenotypic adaptations that enable survival. Such adaptive responses can modulate resistance to other stressors, with stress response systems-such as heat shock proteins and global regulatory networks-conferring cross-protection (Wu et al., 2021). This phenomenon not only enhances bacterial resilience under adverse conditions but also may contribute to the emergence and propagation of antibiotic resistance (Li et al., 2025). Consequently, elucidating the impact of heat stress during food processing on the antibiotic susceptibility of *Salmonella* spp. and *C. jejuni* is critical for guiding evidence-based food safety interventions and limiting the persistence of MDR strains under industrial processing conditions. The objective of the present study was to evaluate the effects of mild and severe heat treatments on antimicrobial resistance in MDR *Salmonella* spp. and *C. jejuni* isolated from raw chicken meat. In addition, the presence of antimicrobial resistance genes was assessed in MDR isolates following thermal exposure.

## Materials and methods

### Multidrug-resistant bacterial isolates

Multidrug-resistant (MDR) *Salmonella* spp. and *C. jejuni* isolates used in the present study were previously recovered from raw chicken meat and fully characterized phenotypically and genotypically in our earlier investigations (Al-Qadiri et al., 2025; Mayyas et al., 2025). Isolates were classified as MDR based on resistance to at least three classes of antimicrobial agents, following the criteria established by the National Antimicrobial Resistance Monitoring System (NARMS, 2017). A total of 44 MDR *Salmonella* spp. isolates and 43 MDR *C. jejuni* isolates were included in this study. Prior to experimentation, bacterial cultures were activated by inoculating *Salmonella* spp. isolates into 100 mL of brain heart infusion (BHI) broth (CM1135B, Oxoid Ltd.) and *C. jejuni* isolates into 100 mL of *Campylobacter* enrichment broth No. 2 (CM0067, Oxoid Ltd.) supplemented with *Campylobacter* growth supplement (SR0232E, Oxoid Ltd.). *Salmonella* cultures were incubated aerobically at 37°C for 24 h, yielding cell densities of approximately 10⁹ CFU/mL. *C. jejuni* cultures were incubated at 37°C for 48 h under microaerophilic conditions (approximately 6–7% O₂) using an anaerobic jar containing CampyGen sachets (CN0025, Oxoid Ltd.), resulting in cell densities of approximately 10⁸ CFU/mL.

Following incubation, 10 mL of each culture was aseptically transferred to sterile centrifuge tubes and centrifuged at 5,000 rpm (3380 × *g*) for 20 min. The resulting cell pellets were washed by resuspension in 50 mL of sterile saline (0.85%, w/v, NaCl) to remove residual growth medium and metabolic byproducts, followed by centrifugation under the same conditions. The washed pellets were then resuspended in 10 mL of sterile saline. Bacterial suspensions were initially standardized spectrophotometrically to a 0.5 McFarland turbidity standard and subsequently verified by viable colony enumeration using the spread plate method on the corresponding culture medium. Based on colony counts, the final standardized inoculum was approximately 10⁸ CFU/mL.

### Heat treatment of bacterial isolates

Heat injury treatments were applied to bacterial cells using thin screw-capped glass tubes prefilled with 9 mL of sterile Mueller–Hinton broth (MHB; CM0405, Oxoid Ltd.), arranged in a rack with adequate spacing to ensure uniform heat distribution. The tubes were submerged in a thermostatically controlled water bath pre-equilibrated to 55, 60, or 85°C, with the water level maintained above the level of the MHB. Temperature was monitored using a digital thermometer placed in an additional tube containing an equivalent volume of MHB. Once the target temperature was attained, each experimental tube was inoculated with 1 mL of the previously standardized bacterial suspension (*ca*. 10⁸ CFU/mL), resulting in a final bacterial concentration of approximately 10⁷ CFU/mL. The inoculated tubes were then maintained at the target temperature in the water bath for the designated exposure period. Tubes were then immediately transferred to an ice–water bath to achieve rapid cooling to 37°C (Al-Qadiri et al., 2008). *Salmonella* spp. isolates were subjected to mild sublethal heat treatments at 55 and 60°C for 5.0 and 1.0 min, respectively, whereas *C. jejuni* isolates were treated at 55 and 60°C for 2.0 and 0.4 min, respectively. The selected time–temperature combinations were based on previously published thermal inactivation studies and our earlier investigations on heat injury in foodborne pathogens (Al-Qadiri et al., 2008). Mild heat treatments (55 and 60°C) were selected to induce controlled sublethal cellular injury while maintaining recoverable bacterial populations for subsequent phenotypic antimicrobial susceptibility testing. In contrast, 85°C for 5 min was included as a lethal thermal treatment to evaluate the persistence of PCR-detectable antimicrobial resistance gene targets following the absence of recoverable culturable cells.

To assess the extent of heat injury and verify the initial and post-treatment bacterial populations, both control (non-heat-treated) and heat-treated samples were enumerated in duplicate using the standard spread plate technique. For *Salmonella* spp., a 1-mL aliquot of each suspension was subdivided into five 0.2-mL portions and plated onto tryptone soya agar (CM0131B, Oxoid Ltd.). Plates were aerobically incubated at 37°C for 24–48 h, and viable cells were enumerated as CFU/mL (Al-Qadiri et al., 2008). For *C. jejuni*, the same spread plate method was applied using *Campylobacter* blood-free agar (CM0739, Oxoid Ltd.) without selective supplement. Plates were incubated at 37°C for 48 h under microaerophilic conditions, and viable cells were enumerated as CFU/mL (Al-Qadiri et al., 2015).

### Antimicrobial susceptibility testing

Antimicrobial susceptibility testing of *Salmonella* spp. was performed using antibiotics representing ten classes commonly employed in the treatment of salmonellosis, following the standard Kirby–Bauer disk diffusion method (Hudzicki, 2009) and in accordance with Clinical and Laboratory Standards Institute guidelines (CLSI, 2020). A 0.1 mL aliquot of the bacterial suspension was evenly streaked onto Mueller–Hinton agar (MHA) plates (CM0337, Oxoid Ltd.), after which antibiotic disks were applied. The inoculated plates were then incubated at 37°C for 18–24 h. The antimicrobial agents, their abbreviations, and disk concentrations (Thermo Fisher Scientific-Oxoid) were as follows: *β*-lactam antibiotics-ampicillin (AMP, 10 μg) and amoxicillin/clavulanic acid (AMC, 20/10 μg); *β*-lactam cephalosporins-ceftazidime (CAZ, 30 μg) and cefotaxime (CTX, 30 μg); aminoglycoside-gentamicin (CN, 10 μg); protein synthesis inhibitors-chloramphenicol (C, 30 μg) and tetracycline (TE, 30 μg); carbapenem-imipenem (IPM, 10 μg); sulfonamide-sulfafurazole (SF, 300 μg); and fluoroquinolone-ciprofloxacin (CIP, 5 μg). All antimicrobial disks were obtained from Thermo Fisher Scientific-Oxoid (CT0003B, CT0223B, CT0412B, CT0166B, CT0024B, CT0013B, CT0054B, CT0455B, CT0075B, and CT0425B, respectively). Isolates were categorized as resistant (R), intermediate (I), or susceptible (S) according to CLSI (2020) guidelines.

Antimicrobial susceptibility testing of *C. jejuni* was conducted using the Kirby–Bauer disk diffusion method, following CLSI-M45 (2015) guidelines for *Campylobacter* spp. and CLSI (2020) criteria for Enterobacterales when species-specific interpretive breakpoints were unavailable. A 0.1 mL aliquot of the bacterial suspension was added to 11 mL of cation-adjusted MHB supplemented with lysed horse blood (Thermo Scientific, CP112-10) and subsequently streaked onto MHA plates containing 5% lysed horse blood. Antibiotic disks were applied to the inoculated plates, which were then incubated under microaerophilic conditions at 42°C for 24 h (CLSI-M45, 2015; Poudel et al., 2022). Twelve classes of antibiotics commonly prescribed for the treatment of campylobacteriosis were used to assess the antimicrobial susceptibility of the isolated *C. jejuni* strains (CLSI-M45, 2015; CLSI, 2020). The antimicrobial agents, their abbreviations, and disk concentrations (Thermo Fisher Scientific-Oxoid) were as follows: *β*-lactam antibiotics-ampicillin (AMP, 10 μg) and amoxicillin/clavulanic acid (AMC, 20/10 μg); *β*-lactam cephalosporins-ceftazidime (CAZ, 30 μg) and cefotaxime (CTX, 30 μg); aminoglycoside-gentamicin (CN, 10 μg); protein synthesis inhibitors-chloramphenicol (C, 30 μg) and tetracycline (TE, 30 μg); macrolides-azithromycin (AZM, 15 μg) and erythromycin (E, 15 μg); quinolone-nalidixic acid (NA, 30 μg); fluoroquinolone-ciprofloxacin (CIP, 5 μg); and sulfonamide-trimethoprim-sulfamethoxazole (SXT, 25 μg). All antimicrobial disks were obtained from Thermo Fisher Scientific-Oxoid (CT0003B, CT0223B, CT0412B, CT0166B, CT0024B, CT0013B, CT0054B, CT0906B, CT0020B, CT0031B, CT0425B, and CT0052B, respectively). *C. jejuni* isolates were classified as resistant (R), intermediate (I), or susceptible (S) according to CLSI (2020) guidelines. Zone diameter breakpoints (mm) for *C. jejuni* were interpreted following CLSI-M45 (2015) for erythromycin, azithromycin, ciprofloxacin, and tetracycline. For antimicrobial agents lacking established *Campylobacter*-specific breakpoints, CLSI (2020) criteria for Enterobacterales were applied.

*Salmonella* spp. and *C. jejuni* isolates displaying resistance to three or more distinct classes of antimicrobials were classified as MDR, in accordance with NARMS (2017). Antimicrobial agents were selected based on their clinical relevance for the treatment of salmonellosis and campylobacteriosis, as well as their documented use in poultry production*.*

### Detection of antimicrobial resistance genes in MDR isolates

Total genomic DNA was extracted from control (non-heat-treated) and heat-treated MDR *Salmonella* spp. and *C. jejuni* isolates using the PureLink™ Genomic DNA Mini Kit (K182002, Thermo Fisher Scientific) according to the manufacturer’s instructions. MDR *Salmonella* spp. isolates were cultured in MHB at 37°C for 18 h, whereas *C. jejuni* isolates were cultured for 48 h at 42°C under microaerophilic conditions in Campylobacter nutrient broth No. 2 (CM0067, Oxoid Ltd.) supplemented with Campylobacter growth supplement (SR0232E, Oxoid Ltd.). The quality and quantity of the extracted DNA were assessed using 1.5% (w/v) agarose gel electrophoresis and a NanoDrop One Microvolume UV-Vis spectrophotometer (Thermo Scientific, Asheville, NC), respectively. PCR amplification was performed using a SimpliAmp™ Thermal Cycler (Thermo Fisher Scientific), and the resulting PCR products were analyzed by 1.5% (w/v) agarose gel electrophoresis and visualized under ultraviolet (UV) light alongside a 100-bp DNA ladder. *Salmonella* isolates were examined by PCR for the presence of genes conferring resistance to *β*-lactams (*bla_TEM_*), tetracycline (*tetB*), and sulfonamides (*sul1* and *sul2*). The primer sequences, expected amplicon sizes, and thermocycling conditions used for amplification of the targeted resistance genes are presented in Table 1. For *C. jejuni*, the resistance genes investigated included *bla_OXA-61_* and *bla_OXA-184_* (*β*-lactams), *aph(2′′)-Ig* and *aph(3′)-IIIa* (aminoglycosides), *tet(O)* (tetracycline), *ermB* (macrolides/erythromycin), and the fluoroquinolone resistance-associated C257T mutation in the *gyrA* gene (Table 2).

**Table 1:** Primers used for the detection of genes encoding antimicrobial resistance and PCR thermocycling conditions among MDR *Salmonella* spp. isolates.

Antimicrobial	Target gene	Forward primer (5′→3′) Reverse primer (5′→3′)	Amplicon (bp)	PCR conditions	Source
*β*-Lactams	*bla_TEM_*	CAGCGGTAAGATCCTTGAGA TTCATCCATAGTTGCCTGACT	661	Denaturation at 94°C for 5 min, followed by 30 cycles of 94°C for 30 s, 55°C for 30 s, 72°C for 50 s, with final extension at 72°C for 7 min	(Li et al. 2013).
Tetracycline	*tet(B)*	GAGACGCAATCGAATTCGG TTTAGTGGCTATTCTTCCTGCC	228	Denaturation at 95°C for 10 min, followed by 35 cycles of 94°C for 45 s, 55-70°C for 50 s, 72°C for 50 s, with final extension at 72°C for 10 min	(Zhu et al. 2017).
Sulfonamide	*Sul1*	CTTCGATGAGAGCCGGCGGC GCAAGGCGGAAACCCGCGCC	437		
	*sul2*	GCGCTCAAGGCAGATGGCATT GCGTTTGATACCGGCACCCGT	285

**Table 2:** Primers used for the detection of genes encoding antimicrobial resistance and PCR thermocycling conditions among MDR *C. jejuni* isolates.

Antimicrobial	Target gene	Forward primer (5′→3′) Reverse primer (5′→3′)	Amplicon (bp)	PCR conditions	Source
*β*-Lactams	*bla_OXA-61_*	CTTTCTCTCCCGCTTCCACT ACCAATTCTTCTTGCCACTTCTTT	203		
	*bla_OXA-184_*	GCTCTCAAGTGCCTGCTTTT AAATCCAACAATCCAAGCCAAA	317		
Aminoglycosides	*aph(2*′′*)-Ig*	GATTTACCTGCCTTGATTCCGG TTCGCCGAAATCTTTCCCA	523	Denaturation at 95°C for 3 min, followed by 35 cycles of 95°C for 30 s, 55°C for 30 s, and 72°C for 60 s, with final extension at 72°C for 5 min.	(Poudel et al., 2022).
	*aph(3*′*)-IIIa*	TGCACTTTGAACGGCATGATG TGTCATACCACTTGTCCGCC	432		
Tetracycline	*tet(O)*	AATATTCAGAGAAAAGGCGGCG GCAGCCATAAAGAACCCCCT	686		
Macrolides-Erythromycin	*ermB*	GGGCATTTAACGACGAAACTGG CTGTGGTATGGCGGGTAAGT	421	Denaturation at 95°C for 3 min, followed by 35 cycles of 95°C for 30 s, 55°C for 30 s, and 72°C for 60 s, with final extension at 72°C for 5 min.	(Cheng et al., 2020).
Fluoroquinolone	*gyrA* (C257T)	GCTCTTGTTTTAGCTGATGCA TTGTCGCCATACCTACAGCTA	620	Denaturation at 95°C for 15 min, followed by 35 cycles of 94°C for 30 s, 56°C for 60 s, and 72°C for 60 s, with final extension at 72°C for 10 min.	(Inglis et al., 2021).

### Statistical analysis

The experiment was conducted in three independent replicate trials. Statistical analyses were performed using SAS software (SAS Institute Inc., Cary, NC, USA; version 8.2). Statistically significant differences (*P* < 0.05) between non-heat-treated and heat-treated isolates with respect to antimicrobial resistance and susceptibility across different antibiotic categories were determined using the Chi-square (χ²) test.

## Results

### Heat-induced changes in phenotypic antimicrobial resistance of MDR *Salmonella* spp.

The phenotypic antimicrobial resistance profiles of MDR *Salmonella* spp. isolates were systematically evaluated against a panel of ten antibiotics before and following exposure to two thermal stress conditions: 55°C for 5.0 min (Table 3) and 60°C for 1.0 min (Table 4). To elucidate the impact of heat stress on resistance phenotypes across diverse antimicrobial classes, shifts in susceptibility categories-resistant, intermediate, and susceptible-were comprehensively analyzed.

**Table 3:** Phenotypic antimicrobial resistance patterns and susceptibility profiles of 44 MDR *Salmonella* spp. isolates after heat treatment at 55 °C for 5.0 min.

Antimicrobials^1^	Control (non-heat-treated)			Heat-treated (55°C, 5.0 min)			Zone diameter breakpoints (nearest whole mm)^2^		
	Resistant (R) No. (%) *	Intermediate (I) No. (%)	Susceptible (S) No. (%) ⁎⁎	Resistant (R) No. (%) *	Intermediate (I) No. (%)	Susceptible (S) No. (%) ⁎⁎	R	I	S
***β-lactams***									
Ampicillin AMP	38 (86.4) ^A^	4 (9.1)	2 (4.5) ^a^	36 (81.8) ^A^	7 (16.0)	1 (2.3) ^a^	< 13	14-16	>17
Amoxicillin/clavulanic acid AMC	22 (50.0) ^A^	12 (27.3)	10 (22.7) ^a^	21 (47.7) ^A^	14 (31.8)	9 (20.5) ^a^	< 13	14-17	> 18
***β-lactam cephalosporin***									
Ceftazidime CAZ	5 (11.4) ^A^	10 (22.7)	29 (66.0) ^a^	2 (4.5) ^B^	10 (22.7)	32 (72.7) ^b^	< 17	18-20	> 21
Cefotaxime CTX	12 (27.3) ^A^	10 (22.7)	22 (50.0) ^a^	10 (22.7) ^A^	11 (25.0)	23 (52.3) ^a^	< 22	23-25	> 26
***Aminoglycoside***									
Gentamicin CN	5 (11.4) ^A^	8 (18.2)	31 (70.5) ^a^	3 (6.8) ^A^	11 (25.0)	30 (68.2) ^a^	< 12	13-14	> 15
***Protein synthesis inhibitors***									
Chloramphenicol C	12 (27.3) ^A^	14 (31.9)	18 (41.0) ^a^	10 (22.7) ^A^	17 (38.6)	17 (38.6) ^a^	< 12	13-17	> 18
Tetracycline TE	30 (68.2) ^A^	9 (20.5)	5 (11.4) ^a^	26 (59.1) ^B^	12 (27.3)	6 (13.6) ^a^	< 11	12-14	> 15
***Beta-lactam carbapenem***									
Imipenem IPM	7 (15.9) ^A^	9 (20.5)	28 (63.6) ^a^	5 (11.4) ^A^	9 (20.5)	30 (68.2) ^a^	<19	20-22	>23
***Sulphonamide***									
Sulphafurazole SF	32 (72.7) ^A^	9 (20.5)	3 (6.8) ^a^	28 (63.6) ^B^	11 (25.0)	5 (11.4) ^a^	< 12	13-16	> 17
***Fluoroquinolone***									
Ciprofloxacin CIP	21 (47.7) ^A^	17 (38.6)	6 (13.6) ^a^	19 (43.2) ^A^	19 (43.2)	6 (13.6) ^a^	<20	21-30	>31

1 Thermo Fisher Scientific-Oxoid.

2 Antimicrobial resistant (R), intermediate (I), and susceptible (S) categories (CLSI, 2020).

⁎ Within the same row, values not sharing the same uppercase letter are significantly different (*P* < 0.05).

⁎⁎ Within the same row, values not sharing the same lowercase letter are significantly different (*P* < 0.05).

**Table 4:** Phenotypic antimicrobial resistance patterns and susceptibility profiles of 44 MDR *Salmonella* spp. isolates after heat treatment at 60 °C for 1.0 min.

Antimicrobials^1^	Control (non-heat-treated)			Heat-treated (60°C, 1.0 min)			Zone diameter breakpoints (nearest whole mm)^2^		
	Resistant (R) No. (%) *	Intermediate (I) No. (%)	Susceptible (S) No. (%) ⁎⁎	Resistant (R) No. (%) *	Intermediate (I) No. (%)	Susceptible (S) No. (%) ⁎⁎	R	I	S
***β-lactams***									
Ampicillin AMP	38 (86.4) ^A^	4 (9.1)	2 (4.5) ^a^	24 (54.5) ^B^	9 (20.5)	11 (25.0) ^b^	< 13	14-16	>17
Amoxicillin/clavulanic acid AMC	22 (50.0) ^A^	12 (27.3)	10 (22.7) ^a^	11 (25.0) ^B^	16 (36.4)	17 (38.6) ^b^	< 13	14-17	> 18
***β-lactam cephalosporin***									
Ceftazidime CAZ	5 (11.4) ^A^	10 (22.7)	29 (66.0) ^a^	2 (4.5) ^B^	10 (22.7)	32 (72.7) ^b^	< 17	18-20	> 21
Cefotaxime CTX	12 (27.3) ^A^	10 (22.7)	22 (50.0) ^a^	5 (11.4) ^B^	15 (34.1)	24 (54.5) ^a^	< 22	23-25	> 26
***Aminoglycoside***									
Gentamicin CN	5 (11.4) ^A^	8 (18.2)	31 (70.5) ^a^	1 (2.3) ^B^	10 (22.7)	33 (75.0) ^a^	< 12	13-14	> 15
***Protein synthesis inhibitors***									
Chloramphenicol C	12 (27.3) ^A^	14 (31.9)	18 (41.0) ^a^	4 (9.1) ^B^	19 (43.2)	21 (47.7) ^b^	< 12	13-17	> 18
Tetracycline TE	30 (68.2) ^A^	9 (20.5)	5 (11.4) ^a^	14 (31.8) ^B^	15 (34.1)	15 (34.1) ^b^	< 11	12-14	> 15
***Beta-lactam carbapenem***									
Imipenem IPM	7 (15.9) ^A^	9 (20.5)	28 (63.6) ^a^	1 (2.3) ^B^	11 (25.0)	32 (72.7) ^b^	<19	20-22	>23
***Sulphonamide***									
Sulphafurazole SF	32 (72.7) ^A^	9 (20.5)	3 (6.8) ^a^	17 (38.6) ^B^	14 (31.8)	13 (29.5) ^b^	< 12	13-16	> 17
***Fluoroquinolone***									
Ciprofloxacin CIP	21 (47.7) ^A^	17 (38.6)	6 (13.6) ^a^	8 (18.2) ^B^	23 (52.3)	13 (29.5) ^b^	<20	21-30	>31

1 Thermo Fisher Scientific-Oxoid.

2 Antimicrobial resistant (R), intermediate (I), and susceptible (S) categories (CLSI, 2020).

⁎ Within the same row, values not sharing the same uppercase letter are significantly different (*P* < 0.05).

⁎⁎ Within the same row, values not sharing the same lowercase letter are significantly different (*P* < 0.05).

#### Phenotypic antimicrobial resistance after heat treatment at 55°C for 5.0 min

Table 3 summarizes the phenotypic antimicrobial resistance profiles of 44 MDR *Salmonella* spp. isolates following exposure to 55°C for 5 min. Overall, this mild thermal treatment exerted only modest effects on resistance patterns across antimicrobial classes. Resistance to ampicillin (AMP) remained high, declining marginally from 86.4% to 81.8%, with susceptibility remaining minimal (2.3%). A similarly non-significant reduction was observed for amoxicillin/clavulanic acid (AMC), where resistance decreased from 50.0% to 47.7%.

Among cephalosporins, resistance to ceftazidime (CAZ) decreased significantly from 11.4% to 4.5% (*P* < 0.05), accompanied by an increase in susceptibility (66.0% to 72.7%), whereas cefotaxime (CTX) resistance remained comparatively stable (27.3% to 22.7%). Gentamicin (CN) resistance showed a slight decline (11.4% to 6.8%), while susceptibility remained consistently high. Tetracycline (TE) resistance decreased significantly following heat exposure (68.2% to 59.1%; *P* < 0.05), and sulphafurazole (SF) resistance was similarly reduced (72.7% to 63.6%; *P* < 0.05). In contrast, chloramphenicol (C) exhibited minimal variation, and no significant changes were observed for ciprofloxacin (CIP) after treatment at 55°C.

#### Phenotypic antimicrobial resistance after heat treatment at 60°C for 1.0 min

In contrast to the 55°C treatment, exposure to 60°C elicited more pronounced reductions in antimicrobial resistance across multiple antibiotic classes. Table 4 presents the phenotypic antimicrobial resistance profiles of 44 MDR *Salmonella* spp. isolates following exposure to 60°C for 1.0 min. Relative to untreated controls, this higher-temperature treatment resulted in substantial declines in resistance frequencies. Resistance to ampicillin (AMP) and amoxicillin/clavulanic acid (AMC) decreased significantly, from 86.4% to 54.5% and from 50.0% to 25.0%, respectively (*P* < 0.05). Among cephalosporins, resistance to both ceftazidime (CAZ) and cefotaxime (CTX) declined, with the reduction reaching statistical significance for CTX. Gentamicin (CN) resistance was also significantly reduced (11.4% to 2.3%; *P* < 0.05), accompanied by a corresponding increase in susceptibility. Significant decreases in resistance were observed for tetracycline (TE), chloramphenicol (C), sulphafurazole (SF), imipenem (IPM), and ciprofloxacin (CIP), with concomitant shifts toward intermediate and susceptible categories (Table 4), underscoring the enhanced impact of elevated thermal stress on antimicrobial resistance phenotypes.

### Molecular detection of ARGs and MDR characteristics of heat-treated *Salmonella* spp.

**Fig. 1:**
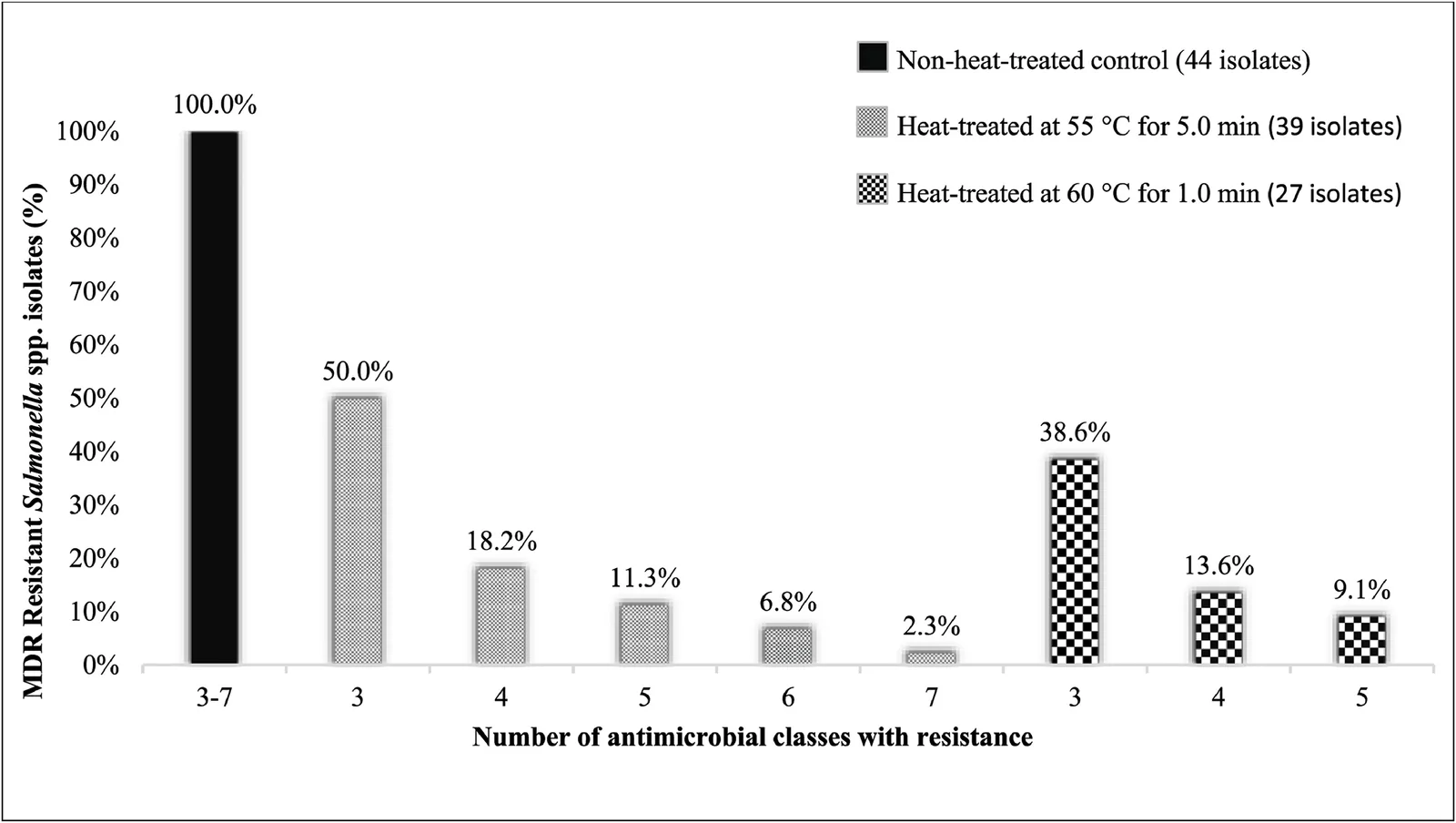
Phenotypic prevalence of antibiotic resistance in MDR *Salmonella* spp. isolates (%).

Table 5 summarizes the distribution of selected antimicrobial resistance genes among MDR *Salmonella* spp. isolates before and after thermal exposure, while Fig. 1 illustrates the distribution of isolates according to the number of antimicrobial classes to which they exhibited resistance. The number of isolates available for post-treatment analysis varied with recovery rates, with fewer viable colonies obtained following exposure to 60°C compared with 55°C.

**Table 5:** Number and percentage of MDR *Salmonella* spp. isolates and suspensions positive for antimicrobial resistance genes after heat treatment.

Resistance genes	Antimicrobials	No. (%) of MDR^1^ isolates positive for antimicrobial resistance genes			No. (%) of suspensions positive for antimicrobial resistance genes
		Non-heat-treated control (*n* = 44)	Heat-treated (55°C, 5.0 min) (*n* = 39)	Heat-treated (60°C, 1.0 min) (*n* = 27)	Heat-treated suspensions (85 °C, 5 min)^2^ (*n* = 44)
	***β*-Lactams**				
*bla_TEM_*		34 (77.3)	31 (79.5)	20 (74.1)	35 (79.5)
	**Tetracycline**				
*tet(B)*		38 (86.4)	33 (84.6)	25 (92.6)	36 (81.8)
					
	**Sulfonamide**				
*Sul1*		15 (34.1)	12 (30.8)	10 (37.0)	14 (31.8)
*sul2*		40 (91.0)	35 (89.7)	23 (85.2)	38 (86.4)

1 MDR: Isolates resistant to three or more antimicrobials.

2 Heat-treated suspensions with no detectable bacterial growth.

Across all experimental conditions, *bla_TEM_* remained highly prevalent, indicating its stability under thermal stress. Similarly, *tet(B)* was consistently detected at high frequencies in both treated and untreated isolates. Among sulfonamide resistance determinants, *sul2* was markedly more prevalent than *sul1* under all conditions examined. Resistance gene detection in heat-treated suspensions (85°C for 5 min), from which no viable bacteria were recovered, yielded frequencies comparable to those observed in viable isolates (Table 5), suggesting persistence of resistance-associated genetic material despite loss of culturability.

Consistent with the observed distribution of resistance determinants, Fig. 1 illustrates that MDR profiles were conserved across experimental conditions, despite a reduction in the number of recoverable MDR isolates with increasing thermal intensity. Following the analysis of MDR *Salmonella* spp., the impact of thermal exposure was further investigated in MDR *C. jejuni* isolates to determine whether comparable heat-induced patterns occurred across bacterial species.

### Heat-induced changes in phenotypic antimicrobial resistance of MDR *C. jejuni*

#### Phenotypic antimicrobial resistance after heat treatment at 55°C for 2.0 min

The phenotypic antimicrobial resistance profiles of MDR *C. jejuni* isolates following mild thermal exposure (55°C for 2.0 min) are presented in Table 6. Overall, heat treatment induced moderate alterations in resistance patterns across multiple antimicrobial classes. Specifically, *β*-lactam resistance declined modestly, with ampicillin (AMP) resistance decreasing from 58.1% in control isolates to 46.5% after treatment, accompanied by a slight increase in susceptibility (30.2% to 34.9%; *P* < 0.05). A similar trend was observed for amoxicillin/clavulanic acid (AMC), where resistance decreased from 11.6% to 4.7%, while susceptibility remained consistently high (83.7–88.4%). Among cephalosporins, resistance to ceftazidime (CAZ) and cefotaxime (CTX) was reduced from 72.1% to 65.1% and from 79.1% to 65.1%, respectively. Gentamicin (CN) exhibited near-complete susceptibility following treatment, increasing from 88.4% in control isolates to 97.7%. Among protein synthesis inhibitors, tetracycline (TE) resistance declined from 60.5% to 46.5%, whereas chloramphenicol (C) showed minimal variation. Macrolides exhibited a modest increase in susceptibility, with reduced resistance to azithromycin (AZM) and erythromycin (E). Likewise, resistance to nalidixic acid (NA) and ciprofloxacin (CIP) decreased from 41.9% to 34.9% and from 46.5% to 37.2%, respectively. Trimethoprim–sulfamethoxazole (SXT) resistance also declined (67.4% to 55.8%), accompanied by an increase in susceptibility (23.3% to 32.6%) (Table 6).

**Table 6:** Phenotypic antimicrobial resistance patterns and susceptibility profiles of 43 MDR *C. jejuni* isolates after heat treatment at 55 °C for 2.0 min.

Antimicrobials^1^	Control (non-heat-treated)			Heat-treated (55°C, 2.0 min)			Zone diameter breakpoints (nearest whole mm)^4^		
	Resistant (R) No. (%) *	Intermediate (I) No. (%)	Susceptible (S) No. (%) ⁎⁎	Resistant (R) No. (%) *	Intermediate (I) No. (%)	Susceptible (S) No. (%) ⁎⁎	R	I	S
***β-Lactams***									
Ampicillin (AMP)^3^	25 (58.1) ^A^	5 (11.6)	13 (30.2) ^a^	20 (46.5) ^B^	8 (18.6)	15 (34.9) ^a^	≤ 13	14-16	≥ 17
Amoxicillin/clavulanic acid (AMC)^3^	5 (11.6) ^A^	2 (4.7)	36 (83.7) ^a^	2 (4.7) ^B^	3 (7.0)	38 (88.4) ^a^	≤ 13	14-17	≥ 18
***β-Lactam cephalosporin***									
Ceftazidime (CAZ)^3^	31 (72.1) ^A^	6 (14.0)	6 (14.0) ^a^	28 (65.1) ^B^	6 (14.0)	9 (21.0) ^b^	≤ 17	18-20	≥ 21
Cefotaxime (CTX)^3^	34 (79.1) ^A^	5 (11.6)	4 (9.3) ^a^	28 (65.1) ^B^	8 (18.6)	7 (16.3) ^b^	≤ 22	23-25	≥ 26
***Aminoglycoside***									
Gentamicin (CN)^3^	2 (4.7) ^A^	3 (7.0)	38 (88.4) ^a^	0 (0.0) ^A^	1 (2.3)	42 (97.7) ^b^	≤ 12	13-14	≥ 15
***Protein synthesis inhibitors***									
Chloramphenicol (C)^3^	3 (7.0) ^A^	2 (4.7)	38 (88.4) ^a^	1 (2.3) ^A^	2 (4.7)	40 (93.0) ^a^	≤ 12	13-17	≥ 18
Tetracycline (TE)^2^	26 (60.5) ^A^	4 (9.3)	13 (30.2) ^a^	20 (46.5) ^B^	7 (16.3)	16 (37.2) ^b^	≤ 22	23-25	≥ 26
***Macrolides***									
Azithromycin (AZM)^2^	4 (9.3) ^A^	3 (7.0)	36 (83.7) ^a^	2 (4.7) ^A^	1 (2.3)	40 (93.0) ^b^	≤12	13-15	≥16
Erythromycin (E)^2^	6 (14.0) ^A^	3 (7.0)	34 (79.0) ^a^	3 (7.0) ^B^	3 (7.0)	37 (86.0) ^b^	≤12	13-15	≥16
***Quinolone***									
Nalidixic acid (NA)^3^	18 (41.9) ^A^	6 (14.0)	19 (44.2) ^a^	15 (34.9) ^B^	9 (21.0)	19 (44.2) ^a^	≤ 13	14-18	≥ 19
***Fluoroquinolone***									
Ciprofloxacin (CIP)^2^	20 (46.5) ^A^	11 (25.6)	12 (28.0) ^a^	16 (37.2) ^B^	11 (25.6)	16 (37.2) ^b^	≤20	21-23	≥24
***Sulfonamide***									
Trimethoprim-sulfamethoxazole (SXT)^3^	29 (67.4) ^A^	4 (9.3)	10 (23.3) ^a^	24 (55.8) ^B^	5 (11.6)	14 (32.6) ^b^	≤10	11-15	≥16

1 Thermo Fisher Scientific-Oxoid.

2 (CLSI-M45, 2015)-*C. jejuni*.

3 (CLSI, 2020)-Enterobacterales.

4 Antimicrobial resistant (R), intermediate (I), and susceptible (S) categories (CLSI, 2020; CLSI-M45, 2015).

⁎ Within the same row, values not sharing the same uppercase letter are significantly different (*P* < 0.05).

⁎⁎ Within the same row, values not sharing the same lowercase letter are significantly different (*P* < 0.05).

#### Phenotypic antimicrobial resistance after heat treatment at 60°C for 0.4 min

In comparison with exposure at 55°C, treatment at 60°C elicited more substantial reductions in antimicrobial resistance across multiple agents. The phenotypic resistance profiles of MDR *C. jejuni* isolates following the second heat treatment (60°C for 0.4 min) are presented in Table 7. Overall, thermal exposure at this higher temperature resulted in pronounced decreases in resistance across several antimicrobial classes.

**Table 7:** Phenotypic antimicrobial resistance patterns and susceptibility profiles of 43 MDR *C. jejuni* isolates after heat treatment at 60 °C for 0.4 min.

Antimicrobials^1^	Control (non-heat-treated)			Heat-treated (60°C, 0.4 min)			Zone diameter breakpoints (nearest whole mm)^4^		
	Resistant (R) No. (%) *	Intermediate (I) No. (%)	Susceptible (S) No. (%) ⁎⁎	Resistant (R) No. (%) *	Intermediate (I) No. (%)	Susceptible (S) No. (%) ⁎⁎	R	I	S
***β-Lactams***									
Ampicillin (AMP)^3^	25 (58.1) ^A^	5 (11.6)	13 (30.2) ^a^	14 (32.6) ^B^	9 (21.0)	20 (46.5) ^b^	≤ 13	14-16	≥ 17
Amoxicillin/clavulanic acid (AMC)^3^	5 (11.6) ^A^	2 (4.7)	36 (83.7) ^a^	0 (0.0) ^B^	6 (14.0)	37 (86.0) ^a^	≤ 13	14-17	≥ 18
***β-Lactam cephalosporin***									
Ceftazidime (CAZ)^3^	31 (72.1) ^A^	6 (14.0)	6 (14.0) ^a^	15 (34.9) ^B^	13 (30.2)	15 (34.9) ^b^	≤ 17	18-20	≥ 21
Cefotaxime (CTX)^3^	34 (79.1) ^A^	5 (11.6)	4 (9.3) ^a^	20 (46.5) ^B^	10 (23.3)	13 (30.2) ^b^	≤ 22	23-25	≥ 26
***Aminoglycoside***									
Gentamicin (CN)^3^	2 (4.7) ^A^	3 (7.0)	38 (88.4) ^a^	0 (0.0) ^A^	0 (0.0)	43 (100.0) ^b^	≤ 12	13-14	≥ 15
***Protein synthesis inhibitors***									
Chloramphenicol (C)^3^	3 (7.0) ^A^	2 (4.7)	38 (88.4) ^a^	0 (0.0) ^B^	4 (9.3)	39 (90.7) ^a^	≤ 12	13-17	≥ 18
Tetracycline (TE)^2^	26 (60.5) ^A^	4 (9.3)	13 (30.2) ^a^	11 (25.6) ^B^	10 (23.3)	22 (51.2) ^b^	≤ 22	23-25	≥ 26
***Macrolides***									
Azithromycin (AZM)^2^	4 (9.3) ^A^	3 (7.0)	36 (83.7) ^a^	1 (2.3) ^B^	5 (11.6)	37 (86.0) ^a^	≤12	13-15	≥16
Erythromycin (E)^2^	6 (14.0) ^A^	3 (7.0)	34 (79.0) ^a^	1 (2.3) ^B^	7 (16.3)	35 (81.4) ^a^	≤12	13-15	≥16
***Quinolone***									
Nalidixic acid (NA)^3^	18 (41.9) ^A^	6 (14.0)	19 (44.2) ^a^	7 (16.3) ^B^	12 (27.9)	24 (55.8) ^b^	≤ 13	14-18	≥ 19
***Fluoroquinolone***									
Ciprofloxacin (CIP)^2^	20 (46.5) ^A^	11 (25.6)	12 (27.9) ^a^	9 (20.9) ^B^	19 (44.2)	15 (34.9) ^a^	≤20	21-23	≥24
***Sulfonamide***									
Trimethoprim-sulfamethoxazole (SXT)^3^	29 (67.4) ^A^	4 (9.3)	10 (23.3) ^a^	14 (32.6) ^B^	12 (27.9)	17 (39.5) ^b^	≤10	11-15	≥16

1 Thermo Fisher Scientific-Oxoid.

2 (CLSI-M45, 2015)-*C. jejuni*.

3 (CLSI, 2020)-Enterobacterales.

4 Antimicrobial resistant (R), intermediate (I), and susceptible (S) categories (CLSI, 2020; CLSI-M45, 2015).

⁎ Within the same row, values not sharing the same uppercase letter are significantly different (*P* < 0.05).

⁎⁎ Within the same row, values not sharing the same lowercase letter are significantly different (*P* < 0.05).

Resistance to *β*-lactams declined significantly, with ampicillin (AMP) resistance decreasing from 58.1% in control isolates to 32.6% after treatment, accompanied by a marked increase in susceptibility (30.2% to 46.5%; *P* < 0.05). Comparable reductions were observed among cephalosporins, where resistance to ceftazidime (CAZ) and cefotaxime (CTX) decreased from 72.1% to 34.9% and from 79.1% to 46.5%, respectively.

Tetracycline (TE) resistance exhibited a marked decline (60.5% to 25.6%), while all isolates were susceptible to gentamicin (CN) following treatment (100% susceptibility). In contrast, macrolides exhibited only modest changes in resistance profiles. Resistance to nalidixic acid (NA) and ciprofloxacin (CIP) decreased substantially, from 41.9% to 16.3% and from 46.5% to 20.9%, respectively. Similarly, trimethoprim–sulfamethoxazole (SXT) resistance was reduced from 67.4% to 32.6% (Table 7), underscoring the enhanced impact of higher thermal intensity on resistance phenotypes.

### Molecular detection of ARGs and MDR characteristics of heat-treated *C. jejuni*

**Fig. 2:**
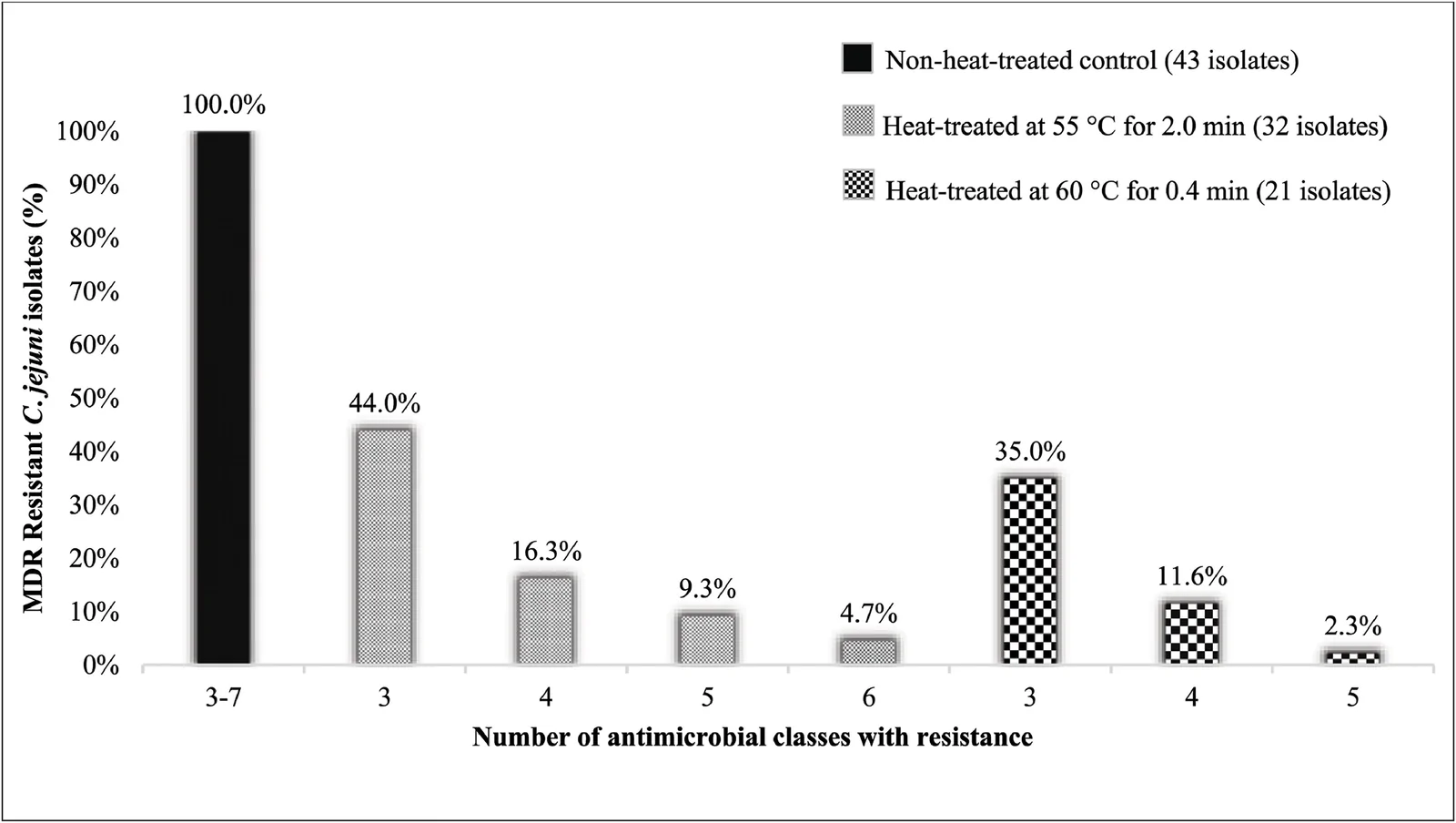
Phenotypic prevalence of antibiotic resistance in MDR *C. jejuni* isolates (%).

The distribution of antimicrobial resistance determinants among MDR *C. jejuni* isolates following thermal exposure is summarized in Table 8, while the phenotypic prevalence of multidrug resistance across antimicrobial classes is illustrated in Fig. 2. Overall, several resistance genes were widely disseminated among the isolates and exhibited notable stability under heat stress. Among *β*-lactam-associated determinants, *bla_OXA-61_*, which confers resistance to *β*-lactam antibiotics such as ampicillin (AMP), was highly prevalent, detected in 81.4% of control isolates and remaining consistently abundant after heat exposure (84.4% at 55°C and 85.7% at 60°C). The *bla_OXA-184_* gene displayed a comparable distribution across treatment conditions.

**Table 8:** Number and percentage of MDR *C. jejuni* isolates and suspensions positive for antimicrobial resistance genes after heat treatment.

Resistance genes	Antimicrobials	No. (%) of MDR^1^ isolates positive for antimicrobial resistance genes			No. (%) of suspensions positive for antimicrobial resistance genes
		Non-heat-treated control (*n* = 43)	Heat-treated (55°C, 2.0 min) (*n* = 32)	Heat-treated (60°C, 0.4 min) (*n* = 21)	Heat-treated suspensions (85 °C, 5 min)^2^ (*n* = 43)
	***β*-Lactams**				
*bla_OXA-61_*		35 (81.4)	27 (84.4)	18 (85.7)	33 (79.1)
*bla_OXA-184_*		30 (69.8)	21 (65.6)	14 (66.7)	27 (65.1)
	**Aminoglycosides**				
*aph(2*′′*)-Ig*		2 (4.7)	1 (3.1)	1 (4.8)	1 (2.3)
*aph(3*′*)-IIIa*		4 (9.3)	2 (6.3)	1 (4.8)	3 (7.0)
	**Tetracycline**				
*tet(O)*		38 (88.4)	26 (81.3)	19 (90.5)	36 (83.7)
					
	**Macrolides-Erythromycin**				
*ermB*		3 (7.0)	2 (6.3)	1 (4.8)	1 (2.3)
					
	**Fluoroquinolone**				
*gyrA* (C257T)		40 (93.0)	29 (90.6)	20 (95.2)	38 (88.4)

1 MDR: Isolates resistant to three or more antimicrobials.

2 Heat-treated suspensions with no detectable bacterial growth.

In contrast, aminoglycoside resistance genes, including *aph(2′)*-*Ig* and *aph*(*3′*)-*IIIa*, which are associated with resistance to agents such as gentamicin (CN), were detected at relatively low frequencies. Conversely, the tetracycline resistance determinant *tet(O)* was highly prevalent (81.3-90.5%) across all conditions. The macrolide resistance gene *ermB*, linked to resistance to erythromycin (E) and azithromycin (AZM), occurred at comparatively low levels.

The fluoroquinolone resistance determinant *gyrA* (C257T), associated with resistance to ciprofloxacin (CIP) and nalidixic acid (NA), exhibited the highest overall prevalence, exceeding 90% across most treatment groups. Furthermore, resistance genes remained detectable in heat-treated suspensions, with *bla_OXA-61_, tet(O)*, and *gyrA* (C257T) persisting as the most frequently identified determinants, underscoring the persistence of PCR-detectable resistance-associated genetic targets despite thermal treatment.

The phenotypic distribution of antimicrobial resistance among MDR *C. jejuni* isolates is illustrated in Fig. 2. Following exposure to 55°C for 2.0 min, 44.0% of isolates were resistant to three classes, while 16.3%, 9.3%, and 4.7% demonstrated resistance to four, five, and six classes, respectively. In contrast, isolates subjected to 60°C for 0.4 min continued to exhibit substantial resistance profiles, with 35.0%, 11.6%, and 2.3% resistant to three, four, and five antimicrobial classes, respectively. These findings underscore the persistence of multidrug resistance despite increased thermal stress, while also highlighting variability in MDR burden in relation to heat treatment intensity.

## Discussion

Previous investigations by our group have systematically characterized AMR in foodborne pathogens associated with poultry products. MDR *Salmonella* spp. isolated from raw chicken meat exhibited extensive resistance to multiple clinically important antimicrobial classes, accompanied by the presence of key resistance determinants (Al-Qadiri et al., 2025). In parallel, recent surveillance of *C. jejuni* from retail poultry meat revealed a high prevalence of resistant isolates, including marked resistance to *β*-lactams, tetracyclines, and fluoroquinolones, underpinned by the widespread distribution of resistance-associated genetic determinants such as *bla_OXA-61_, tet(O)*, and *gyrA* (C257T) (Mayyas et al., 2025). Building on this foundation, the present study reveals a pronounced discordance between phenotypic AMR and corresponding genotypic determinants in MDR *Salmonella* spp. and *C. jejuni* subjected to thermal stress within a chicken meat matrix. Heat exposure resulted in a measurable attenuation of phenotypic resistance across multiple antimicrobial classes, with increasingly pronounced effects observed at elevated temperatures.

Our findings demonstrate a clear temperature-dependent pattern in antimicrobial resistance responses. While moderate thermal exposure at 55°C resulted in only limited alterations in phenotypic resistance profiles (Table 3, Table 6), increasing the treatment temperature to 60°C produced a pronounced and statistically significant reduction in resistance across multiple antimicrobial classes. In *Salmonella* spp., resistance to ampicillin declined markedly from 86.4% to 54.5%, and to amoxicillin/clavulanic acid from 50.0% to 25.0%, accompanied by concurrent decreases in resistance to cephalosporins, aminoglycosides, and tetracyclines (Table 4). A comparable trend was observed in *C. jejuni*, where tetracycline resistance decreased from 60.5% to 25.6% (Table 7).

These phenotypic changes were not accompanied by corresponding alterations in the molecular detection of ARGs, which remained consistently detectable at high prevalence across all experimental conditions, including suspensions subjected to lethal heat treatment (85°C for 5 min). In *Salmonella* spp., *bla_TEM_, tet(B)*, and *sul2* were detected at high frequencies (Table 5), while *gyrA* (C257T), *tet(O)*, and *bla_OXA-61_* remained detectable in *C. jejuni* (Table 8). ARG targets remained detectable in heat-treated samples (85 °C) despite the absence of culturable bacterial cells, indicating the persistence of amplifiable DNA fragments following severe thermal exposure. These findings are consistent with previous reports demonstrating that thermal processing can effectively eliminate bacterial culturability while leaving residual DNA targets sufficiently intact for PCR-based detection, thereby highlighting the potential discrepancy between culture-dependent assessments of bacterial survival and molecular detection of genetic markers (Li et al., 2013; Chen et al., 2022; Baymiev et al., 2020). However, PCR detection alone does not provide information regarding DNA integrity, biological functionality, expression, or transferability of the detected genetic material (James et al., 2021). Accumulating evidence indicates that environmental stressors encountered along the food production continuum, including temperature fluctuations during processing, can modulate antimicrobial susceptibility by reshaping bacterial physiology and triggering adaptive stress-response pathways (Mc Mahon et al., 2007; Al-Nabulsi et al., 2011; Wu et al., 2021; Li et al., 2025).

The observed discordance between phenotypic resistance and genotypic profiles constitutes a pivotal finding of this study, underscoring the persistence of PCR-detectable resistance gene targets even following effective thermal inactivation of bacterial cells (Macesic et al., 2025; Li et al., 2025). The observed attenuation of phenotypic antimicrobial resistance may be attributable to heat-induced disruption of essential cellular structures and resistance-associated proteins. At elevated temperatures (≥ 60 °C), bacterial responses appear to transition from adaptive stress responses to irreversible cellular injury, primarily as a consequence of the denaturation of essential metabolic proteins and the consequent impairment of critical cellular functions, as reported by Nguyen et al. (2006). This disruption may impair protein synthesis, including the production of resistance-associated proteins, thereby contributing to the pronounced reduction in phenotypic antimicrobial resistance observed in the present study. In contrast, DNA exhibits greater thermal stability than many cellular proteins and other structural components, allowing at least some PCR-amplifiable resistance gene targets to remain detectable despite the loss of bacterial viability and phenotypic resistance. Accordingly, the observed attenuation of phenotypic resistance following thermal treatment is consistent with a transition from early adaptive stress responses to irreversible cellular injury induced by heat exposure (Zhang and Gross, 2021; Wesche et al., 2009). However, exposure to sublethal heat stress may trigger adaptive cellular responses, including the induction of heat shock proteins (HSPs), which act as molecular chaperones to stabilize protein structures, prevent misfolding and aggregation, and support the maintenance of critical cellular functions under thermal stress (Zhang and Gross, 2021; Wesche et al., 2009). In particular, the ClpL chaperone has been shown to stabilize cell wall-associated proteins, potentially reducing antimicrobial penetration and transiently enhancing cellular tolerance during stress exposure (Tran et al., 2011). Nevertheless, prolonged or intensified thermal stress may overwhelm these protective mechanisms, resulting in irreversible damage to membrane integrity, impaired protein function, and disruption of cellular homeostasis. Such heat-induced alterations may increase antibiotic susceptibility by enhancing antimicrobial penetration and compromising resistance-associated cellular processes.

The persistence of PCR-detectable ARG targets following severe thermal exposure may be attributable to the greater thermal stability of DNA relative to proteins and other cellular constituents. Thermal processing is known to induce extensive damage to bacterial cell membranes, ribosomes, enzyme systems, and other essential structures required for cellular viability and metabolic activity. Consequently, bacterial cells may lose viability and exhibit reduced phenotypic antimicrobial resistance, whereas amplifiable DNA targets remain detectable by conventional PCR. This interpretation is consistent with the marked reduction in phenotypic resistance observed despite the continued detection of the selected ARG targets. Importantly, the present study did not evaluate DNA integrity, transcriptional activity, plasmid stability, or horizontal gene transfer potential. Therefore, the persistence of PCR-detectable ARG targets following thermal treatment should be interpreted as evidence of the persistence of amplifiable DNA rather than as evidence of biologically functional resistance determinants or their potential for dissemination (Oliver, 2010; Zhao et al., 2017; Zhang and Lu, 2023). DNA generally exhibits greater thermal stability than proteins and many other cellular constituents, particularly within complex food matrices such as meat, where matrix components may provide physical protection against heat-induced degradation (Montowska and Pospiech, 2010). The composition of the food matrix is therefore an important determinant of microbial thermal inactivation and the persistence of ARG targets. In chicken meat, proteins and lipids may partially reduce heat transfer efficiency and enhance the thermal protection of both bacterial cells and their associated genetic material (Huang et al., 2019; Tian et al., 2017). Collectively, these factors may contribute to the continued detection of PCR-amplifiable ARG targets following thermal processing.

Comparative analysis indicated that *C. jejuni* exhibited greater thermal sensitivity than *Salmonella* spp., highlighting distinct physiological differences in heat tolerance between these two foodborne bacterial genera. This observation is consistent with previous reports describing the lower thermal resistance of *Campylobacter* spp. compared with *Salmonella* spp. (de Jong et al., 2012). In *Salmonella* spp., although heat treatment resulted in a substantial reduction in tetracycline resistance (Table 4), the prevalence of the associated resistance determinant *tet(B)* remained consistently high across all treatment conditions (Table 5). Similarly, *bla_TEM_* and *sul2* remained highly prevalent following thermal exposure. The persistence of *tet(B), bla_TEM_*, and *sul2* after heat treatment likely reflects the stability of detectable genetic material rather than the maintenance of functional resistance mechanisms. Molecular detection alone does not confirm gene expression, protein activity, or phenotypic resistance, particularly following stress conditions that may compromise cellular integrity and physiological functions. Although *tet(B)* encodes an efflux pump implicated in tetracycline resistance, the presence of this gene alone does not confirm the preservation of gene expression, protein functionality, or the associated resistance phenotype following thermal stress. Thus, PCR-based detection reflects the persistence of amplifiable genetic material, whereas phenotypic antimicrobial resistance is dependent on the structural integrity, expression, and functionality of the bacterial cellular systems involved in resistance mechanisms. A comparable genotype-phenotype discordance was observed in *C. jejuni*. Despite substantial reductions in antimicrobial resistance following thermal treatment, the prevalence of resistance-associated genetic markers, including the *gyrA* (C257T) mutation and *tet(O)*, remained largely unchanged across the tested conditions (Table 8). Notably, fluoroquinolone resistance in *C. jejuni* is primarily mediated by specific mutations within the quinolone resistance-determining region (QRDR) of the *gyrA* gene rather than by the mere presence of the gene itself. Therefore, PCR detection of the *gyrA* (C257T) mutation should be interpreted as evidence of the persistence of a resistance-associated genetic determinant, but not as direct confirmation of an active fluoroquinolone-resistant phenotype. These findings highlight the importance of integrating molecular and phenotypic approaches when assessing the impact of thermal processing on antimicrobial resistance. These findings indicate that the mere presence of resistance genes does not necessarily correlate with phenotypic expression under stress conditions. Consequently, reliance solely on conventional culture-based assays may not fully capture the persistence of PCR-detectable resistance gene targets in thermally processed food products. Collectively, the present findings demonstrate that thermal processing substantially reduces phenotypic antimicrobial resistance in MDR *Salmonella* spp. and *C. jejuni*, while selected resistance gene targets remain detectable by conventional PCR following heat exposure. These results highlight the importance of interpreting phenotypic and molecular findings as complementary rather than interchangeable indicators of antimicrobial resistance. While thermal treatment effectively compromises bacterial viability through extensive cellular damage, detectable DNA sequences may persist under the conditions examined. Additional studies incorporating quantitative molecular approaches, DNA integrity assessments, and gene expression analyses are required to clarify the biological significance of these residual genetic signals following thermal processing.

A limitation of the present study is that conventional PCR confirms only the presence of amplifiable ARG targets and does not provide information regarding DNA integrity, transcriptional activity, plasmid stability, or horizontal gene transfer potential. Accordingly, the persistence of PCR-detectable ARG targets following thermal treatment should be interpreted as evidence of the persistence of amplifiable DNA rather than as evidence of biologically functional resistance determinants or their dissemination potential. Furthermore, because antimicrobial susceptibility was assessed immediately after thermal exposure, the observed phenotypic changes may reflect transient physiological impairment rather than stable alterations in antimicrobial resistance. Future studies incorporating post-recovery/passaging experiments, together with complementary molecular approaches such as quantitative gene expression analyses, are warranted to determine whether these phenotypic changes persist following recovery from thermal stress and to further elucidate the biological significance of PCR-detectable ARGs after thermal processing.

## Conclusion

This study demonstrates that thermal processing differentially affects phenotypic antimicrobial resistance and PCR-detectable antimicrobial resistance gene targets in multidrug-resistant *Salmonella* spp. and *Campylobacter jejuni*. Thermal treatment resulted in a marked reduction in recoverable bacterial populations and an apparent attenuation of phenotypic antimicrobial resistance. However, because antimicrobial susceptibility was assessed immediately after heat exposure, the observed reduction may reflect transient physiological impairment associated with sublethal heat injury rather than a stable loss of antimicrobial resistance mechanisms.

In contrast, the selected ARG targets remained consistently detectable following thermal treatment, including under conditions in which no culturable bacteria were recovered. These findings most likely reflect the persistence of PCR-amplifiable DNA, attributable to the greater thermal stability of DNA relative to other cellular constituents, and should not be interpreted as evidence that the detected resistance determinants remained biologically functional. Because conventional PCR does not provide information regarding DNA integrity, transcriptional activity, plasmid stability, or horizontal gene transfer potential, the continued detection of ARG targets should not be interpreted as evidence of functional resistance or dissemination potential.

Overall, the findings may highlight the value of integrating phenotypic antimicrobial susceptibility testing with molecular detection approaches to improve the interpretation of antimicrobial resistance dynamics in thermally processed foods. They also underscore the limitations of conventional culture-based and PCR-based methods in determining the biological fate and functional significance of resistance determinants following heat exposure. Future studies incorporating post-recovery/passaging experiments together with quantitative molecular, viability-based, and functional analyses are warranted to determine the stability of heat-induced phenotypic changes and to further elucidate the biological significance of PCR-detectable ARGs following thermal processing.

## Funding

No external funding was received to support this work.

## Author contribution

**Hamzah M. Al-Qadiri** contributed to the study design and supervision, conducted experimental investigations and PCR verification, evaluated the findings, and drafted the manuscript. **Batool Khataybeh** participated in the experimental investigations and contributed to the development of the written manuscript. **Amal Mayyas** contributed to the formulation of the research concept and development of the manuscript. **Murad A. Al-Holy** contributed to establishing the research concept and providing scientific oversight throughout the study. **Amin N. Olaimat** contributed to the quantitative assessment of the results and evaluation of the study outcomes. **Islam Hamad** contributed to the examination of the results and their scientific assessment. **Nivin Al-Alami** contributed to the examination and verification of the PCR data and assessment of the accuracy of the molecular findings. **Faris Ghalib Bakri** participated in the experimental investigations and evaluated the resulting data. **Hana Zakaria** provided scientific oversight and contributed to the assessment of the experimental results. **Mohmmad Al-Qaisi** provided scientific guidance and contributed to the evaluation of the study results. **Tariq N. Alsmairat** contributed to the scientific assessment and interpretation of the research outcomes. **George Y. Nijmeh**contributed to the evaluation and interpretation of the study results. **Rana Al-Akhras** contributed to the microbiological culturing, evaluation of the isolates, and assessment of the microbiological findings. **Barbara A. Rasco** contributed to the formulation of the research concept and development of the manuscript. **Zeinab M.H. Mahasneh** participated in the experimental investigations and contributed to the scientific development of the manuscript. All authors have approved the content, fulfilled the authors' criteria, and contributed significantly to this research.

## Disclosures

The authors declare that they have no known competing financial interests or personal relationships that could have appeared to influence the work reported in this article.

## Data Availability

Data will be provided upon request.
